# Dynamic causal modelling of evoked potentials: A reproducibility study

**DOI:** 10.1016/j.neuroimage.2007.03.014

**Published:** 2007-07-01

**Authors:** Marta I. Garrido, James M. Kilner, Stefan J. Kiebel, Klaas E. Stephan, Karl J. Friston

**Affiliations:** The Wellcome Dept. of Imaging Neuroscience, University College London, Queen Square, London, WC1N 3BG, UK

## Abstract

Dynamic causal modelling (DCM) has been applied recently to event-related responses (ERPs) measured with EEG/MEG. DCM attempts to explain ERPs using a network of interacting cortical sources and waveform differences in terms of coupling changes among sources. The aim of this work was to establish the validity of DCM by assessing its reproducibility across subjects. We used an oddball paradigm to elicit mismatch responses. Sources of cortical activity were modelled as equivalent current dipoles, using a biophysical informed spatiotemporal forward model that included connections among neuronal subpopulations in each source. Bayesian inversion provided estimates of changes in coupling among sources and the marginal likelihood of each model. By specifying different connectivity models we were able to evaluate three different hypotheses: differences in the ERPs to rare and frequent events are mediated by changes in forward connections (F-model), backward connections (B-model) or both (FB-model).

The results were remarkably consistent over subjects. In all but one subject, the forward model was better than the backward model. This is an important result because these models have the same number of parameters (i.e., the complexity). Furthermore, the FB-model was significantly better than both, in 7 out of 11 subjects. This is another important result because it shows that a more complex model (that can fit the data more accurately) is not necessarily the most likely model. At the group level the FB-model supervened. We discuss these findings in terms of the validity and usefulness of DCM in characterising EEG/MEG data and its ability to model ERPs in a mechanistic fashion.

## Introduction

In dynamical causal modelling, one views the brain as a dynamic network of interacting sources that produces observable responses. This perspective furnishes spatiotemporal, generative or forward models for evoked responses as measured with EEG/MEG (David et al., 2005; Friston et al., 2003). In brief, DCM entails specification of a plausible model of electrodynamic responses. This model is inverted by optimising a variational free energy bound on the model evidence to provide the conditional density of the model parameters and the models evidence for model comparison. This is an important advance over conventional analyses of evoked responses because it places natural constraints on the inversion; namely, activity in one source has to be caused by activity in another. We have focused on establishing the face-validity of DCM for ERPs (see David et al., 2005, 2006; Kiebel et al., 2006). In this work, we address predictive-validity in terms of the reproducibility of DCM over subjects. In brief, if DCM discloses true underlying brain network, then the results of model comparison in one subject should predict the results in another (i.e., they should be consistent over subjects). To ensure we engaged the same network in all subjects, we used a mismatch negativity (MMN) paradigm. This represents one of the most well studied and reproducible phenomena in ERP research (Näätänen et al., 2001).

### Dynamic causal modelling

Previous work suggests that event-related responses can be modelled as perturbations of cortical networks (David et al., 2005; Jansen and Rit, 1995). In particular, we have shown that dynamic causal models (DCMs) can explain event-related potentials (ERPs) and fields (ERFs) measured with electroencephalography (EEG) and magnetoencephalography (MEG), respectively. Furthermore, David et al. (2006) showed that differences in evoked responses can be explained by changes in effective connectivity or coupling among neuronal sources. DCM can be regarded as an elaboration of conventional spatial forward models of EEG/MEG data, in which the sources are coupled according to biological constraints. The inversion of a DCM provides information about the underlying cortical pathways and their causal architecture. In this work, we accessed the reproducibility of DCM by testing hypotheses about cortical networks suggested by a predictive coding view of novelty or mismatch responses. Predictive coding is a formulation of perceptual learning and inference that rests on hierarchical Bayes (Rao and Ballard, 1999; Friston, 2003). Critically, by formulating different implementations of perceptual learning in terms of different DCMs we were able to adjudicate among competing hypotheses using Bayesian model comparison. We were then able to assess the reproducibility of model comparison over subjects.

Bayesian model comparison (Penny et al., 2004) selects the model, among competing models, that best explains the data. Given equal prior probabilities for the models considered, they are compared using their marginal likelihood or evidence for each model. In this work, we assessed the predictive validity of DCM by testing its reproducibility in a multi-subject study, under an auditory oddball paradigm. Specifically, we looked at reproducibility, in terms of model comparison, from subject to subject. The choice of the oddball paradigm was motivated by the large body of work in this field and current interest in the mechanisms underlying the generation of the MMN; in particular, the specific hypotheses about these mechanisms furnished by theoretical treatments (Winkler et al., 1996; Friston, 2005).

### Mismatch responses

Small changes in the acoustic environment initiate involuntary attentional capture, which may be engaged automatically by an auditory change detection mechanism indexed by the MMN. It has been proposed that a temporo-prefrontal network generates the MMN by comparing sensory input with the memory trace of previous stimuli. Novel events (oddballs) embedded in a stream of repeated or familiar events (standards) produce a distinct response that can be recorded non-invasively with electrophysiological techniques such as EEG and MEG. The MMN is the negative component of the waveform obtained by subtracting the event-related response to a standard from the response to an oddball, also called a deviant in the literature. This pre-attentive response, to sudden changes in stimulation, peaks at ≈ 100–200 ms from change onset; with the greatest intensity located over the frontal region (channels Fz and FCz). Given its automatic nature, the MMN has been associated with pre-attentive cognitive operations in audition and has, for this reason, been proposed as a reflection of ‘primitive intelligence’ in the auditory cortex (Näätänen et al., 2001).

There have been several compelling mechanistic accounts of how the MMN might arise, many focussing on plasticity or adaptation at a synaptic and neuronal level (e.g. May et al., 1999). Winkler et al. (1996) suggested that the MMN might arise from a model-adjustment process, whereby the auditory system adjusts its perceptual model to adapt to the stimuli encountered. A similar conclusion was reached from a theoretical treatment of perceptual inference and learning based on hierarchical Bayes (Friston, 2005). In this framework, evoked responses correspond to prediction error that is explained away (within trial) by self-organising neuronal dynamics during perception and is suppressed (between trials) by changes in synaptic efficacy during learning. The suppression of evoked responses, to a repeated event, is a ubiquitous phenomenon in neuroscience. It is seen at the level of single-unit responses (where it is referred to as repetition suppression; Desimone, 1996) and is a long-standing observation in human neuroimaging (where it is often referred to as adaptation, e.g., cerebellar adaptation during motor repetitions; Friston et al., 1992 or repetition effects in visual studies; Henson et al., 2003).

In the present context, changes in synaptic connections, during the repeated presentation of standards may render suppression of prediction error more efficient. This would lead to a reduction in evoked responses and the emergence of a mismatch response, when an unlearned stimulus is presented. In the theoretical accounts this differential response is mediated by differences in effective connectivity or coupling between cortical levels. In short, mechanistic theories about the MMN posit changes in synaptic efficacy that, in a hierarchical setting, may involve forward and/or backward extrinsic (between cortical sources) connections or intrinsic (local) connectivity. In what follows, we use DCM and model comparison to ask whether differences in ERPs to frequent and rare events are better explained by changes in forward connections, backward connections or both.

## Materials and methods

### Dynamic causal modelling

Most approaches to connectivity in the MEG/EEG literature use functional connectivity measures, such as phase-synchronisation, temporal correlations or coherence, to establish statistical dependencies between activities in two sources. Although functional connectivity can be used to establish a statistical dependency it does not provide information about the causal architecture of the interactions. DCM uses the concept of effective connectivity, as opposed to functional connectivity. Effective connectivity refers explicitly to the influence one neuronal system exerts over another. This influence is parameterised in a causal model, which can then be estimated using model inversion. In DCM, the brain is regarded as a deterministic nonlinear dynamic system that is subject to inputs and produces outputs (Friston et al., 2003). Effective connectivity is estimated by perturbing the system and measuring the response using Bayesian model inversion. DCM provides an account of the interactions among cortical regions and allows one to make inferences about system parameters and investigate how these parameters are influenced by experimental factors. Furthermore, by taking the marginal likelihood over the conditional density of the model parameters, one can estimate the probability of the data, given a particular model. This is known as the marginal likelihood or evidence and can be used to compare different models.

#### Hierarchical MEG/EEG neural mass models

DCMs for MEG/EEG use neural mass models (David and Friston, 2003) to explain source activity in terms of the ensemble dynamics of the interacting inhibitory and excitatory subpopulations of neurons, based on the model of Jansen and Rit (1995). This model emulates the activity of a source using three neural subpopulations, each assigned to one of three cortical layers; an excitatory subpopulation in the granular layer, an inhibitory subpopulation in the supra-granular layer and a population of deep pyramidal cells in the infra-granular layer. The excitatory pyramidal cells receive excitatory and inhibitory input from local interneurons (via intrinsic connections, confined to the cortical sheet), and send excitatory outputs to remote cortical areas via extrinsic connections. David et al. (2005) describe a hierarchical model using extrinsic connections among multiple sources that conform to the connectivity rules described in (Felleman and Van Essen, 1991). These rules allow one to build a network of coupled sources linked by extrinsic connections. Within this model, bottom-up or forward connections originate in the infra-granular layers and terminate in the granular layer; top-down or backward connections link agranular layers and lateral connections originate in infra-granular layers and target all layers. All these extrinsic cortico-cortical connections are excitatory and are mediated through the axons of pyramidal cells. Exogenous inputs to the model have the same characteristics as forward connections and deliver exogenous (i.e., sensory) input, *u*, to the granular layer.

The DCM is specified in terms of some state equations that summarise the average synaptic dynamics in terms of spike-rate-dependent current and voltage changes, for each subpopulation

(1) $$\overset{\cdot }{x}=f(x,u,\theta )$$

This means that the evolution of the neuronal state *x* is a function (parameterised by *θ*) of the state and the input. An output equation couples specific states (the average depolarisation of pyramidal cells in each source), *x*_0_, to the MEG/EEG signals, *y*, using a conventional linear electromagnetic forward model.

(2) $$y=L(\theta )x_{0}+\varepsilon$$

Eq. (1) summarises the state equations specifying the rate of change of the potentials as a function of the current and how currents change as a function of the currents and the potentials (see David et al., 2005, 2006; David and Friston, 2003 for details). The state equations embody the connection rules described above, where *θ* includes the parameters for forward, backward and lateral connections and their modulation. These are the parameters we want to estimate. Eq. (2) links the neuronal states to observed channel data. In this application, the lead field *L*(*θ*) was parameterised in terms of the location and orientation of each source as described in Kiebel et al. (2006).

#### Bayesian model comparison

Inversion of a specific DCM, *m*, corresponds to approximating the posterior probability on the parameters, which is proportional to the probability of the data (the likelihood) conditioned upon the model and its parameters, times the prior probability on the parameters

(3) p(θ|y,m)∝p(y|θ,m)p(θ|m)

This approximation uses variational Bayes that is formally identical to Expectation–Maximisation (EM), as described in Friston (2002). The EM can be formulated in analogy to statistical mechanics as a gradient descent on the free energy, *F*, of a system. The aim is to minimise the free energy with respect to a variational density *q*(*θ*). When the free energy is minimised *q*(*θ*) = *p*(*θ*|*y*,*m*), the free energy *F* = − ln *p*(*y*|*m*) is the negative marginal log-likelihood or negative log-evidence. After convergence and minimisation of the free energy, the variational density is used as an approximation to the desired conditional density and the log-evidence is used for model comparison.

One often wants to compare different models and select the best before making statistical inferences on the basis of the conditional density. The best model, given the data, is the one with highest log-evidence ln *p*(*y*|*m*) (assuming a uniform prior over models). Given two models *m*_1_ and *m*_2_ one can compare them by computing their Bayes factor (Penny et al., 2004) or, equivalently, the difference in their log-evidences ln *p*(*y*|*m*_1_) − ln *p*(*y*|*m*_2_). If this difference is greater than about three (i.e., their relative likelihood is more then 20:1) then one asserts there is strong evidence in favour of the first model.

The formalism described above is suitable for comparing different models of a given data set, for instance data acquired from a single subject. However, one may wish to select the model that best explains multiple data sets, i.e., the best model at the group level. Assuming each data set is independent of the others (i.e., all subjects are independent of each other), we can simply multiply the marginal likelihoods, or, equivalently, add the log-evidences from each subject

(4) $$\ln p(y_{1},\ldots ,y_{n}|m_{i})=\sum_{j=1}^{n}\ln p(y_{j}|m_{i})$$

to obtain the log-evidence for the *i*th model across all *n* subjects.

#### Parameter estimation at the group level

Bayes’ theorem updates our belief about a parameter in the light of new evidence from the data. Bayesian inference can be particularly useful at the second (between-subject) level of statistical analysis (Neumann and Lohmann, 2003). In particular, it is easy to combine the conditional densities from several subjects to obtain a single conditional density for the group. The conditional probability of the parameters given data from all subjects is

(5) $$p(\theta |y_{1},\ldots ,y_{n})\propto p(y_{1},\ldots ,y_{n}|\theta )p(\theta )$$

If the conditional densities for each subject *p*(*θ*_*j*_|*y*_*j*_) = *N*(*μ*_*j*_,*Σ*_*j*_) have a Gaussian form, the mean *μ* and the precision *Λ* = *Σ*^− 1^ of the conditional density of the parameters at the group level can be calculated from the individual mean *μ*_*j*_ and precision matrices *Λ*_*j*_ = *Σ*_*j*_^− 1^.

(6) $$\mu =\Lambda^{-1}\sum_{j=1}^{n}\Lambda_{j}\mu_{j}$$

$$\Lambda =\sum_{j=1}^{n}\Lambda_{j}$$

This provides a useful way to summarise the results of several DCMs from different subjects.

### Experimental design

#### Subjects

We studied a group of 13 healthy volunteers aged 24–35 (5 female). Each subject gave signed informed consent before the study, which proceeded under local ethical committee guidelines.

#### Task

Subjects sat on a comfortable chair in front of a desk in a dimly illuminated room. Electroencephalographic activity was measured during an auditory ‘oddball’ paradigm, in which subjects heard of “standard” (1000 Hz) and “deviant” tones (2000 Hz), occurring 80% (480 trials) and 20% (120 trials) of the time, respectively, in a pseudo-random sequence. The stimuli were presented binaurally via headphones for 15 min every 2 s. The duration of each tone was 70 ms with 5 ms rise and fall times. The subjects were instructed not to move, to keep their eyes closed and to count the deviant tones.

### Data acquisition and processing

EEG was recorded with a *Biosemi* system with 128 scalp electrodes. Data were recorded at a sampling rate of 512 Hz. Vertical and horizontal eye movements were monitored using EOG (electro-oculograms) electrodes. The data were epoched offline, with a peri-stimulus window of − 100 to 400 ms, down-sampled to 200 Hz, band-pass filtered between 0.5 and 40 Hz and re-referenced to the average of the right and left ear lobes. Trials in which the absolute amplitude of the signal exceeded 100 μV were excluded. Two subjects were eliminated from further analysis due to excessive trials containing artefacts. In the remaining subjects, an average 18% of trials were excluded. For computational expediency the dimensionality of the data was reduced to three channel mixtures or spatial modes. These were the principal modes of a singular value decomposition of the channel data between 0 and 250 ms, from both trial types. The use of three principal eigenvariates preserved more than 73% of the variance in all subjects.

### DCM specification

In this section we specify three plausible models defined by a given architecture and dynamics, which we use here to test the validity of DCM.

The network architecture was motivated by recent electrophysiological and neuroimaging studies looking at the sources underlying the MMN (Opitz et al., 2002; Doeller et al., 2003). We assumed five sources, modelled as equivalent current dipoles (ECDs), over left and right primary auditory cortices (A1), left and right superior temporal gyrus (STG) and right inferior frontal gyrus (IFG). Our mechanistic model attempts to explain the generation of each individual response (i.e., responses to standards and responses to deviants). Therefore, left and right A1 were chosen as cortical input stations for processing the auditory information. Opitz et al. (2002) identified sources for the differential response, with fMRI and EEG measures, in both left and right STG, and right IFG. Here we employ the coordinates reported by Opitz et al. (2002) (for left and right STG and right IFG) and Rademacher et al. (2001) (for left and right A1) as prior source location means, with a prior variance of 32 mm. We converted these coordinates, given in the literature in Talairach space, to MNI space using the algorithm described in http://imaging.mrc-cbu.cam.ac.uk/imaging/MniTalairach. The moment parameters had prior mean of 0 and a variance of 8 in each direction. We have used these parameters as priors to estimate, for each individual subject, the posterior locations and moments of the ECDs (Table 1).

Using these sources and prior knowledge about the functional anatomy we constructed the following DCM: An extrinsic input entered bilaterally to primary auditory cortex (A1), which were connected to their ipsilateral STG. Right STG was connected with the right IFG. Inter-hemispheric (lateral) connections were placed between left and right STG. All connections were reciprocal (i.e., connected with forward and backward connections or with bilateral connections).

**Fig. 1:**
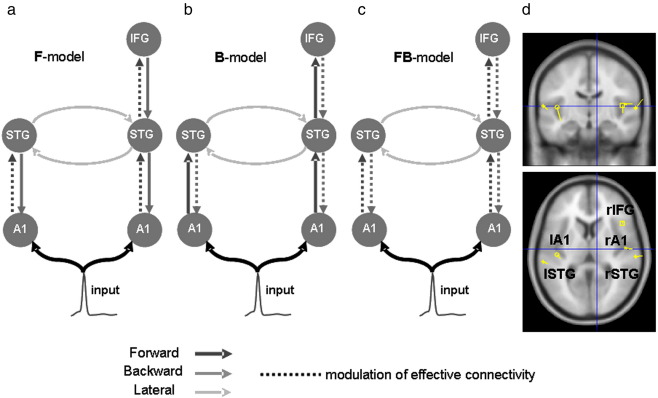
Model specification. The sources comprising the network are connected with forward (dark grey), backward (grey) or lateral (light grey) connections as shown. A1: primary auditory cortex, STG: superior temporal gyrus, IFG: inferior temporal gyrus. Three different models were tested within the same architecture (a–c), allowing for learning-related changes in forward F, backward B and forward and backward FB connections, respectively. The broken lines indicate the connections we allowed to change. Sources of activity, modelled as dipoles (estimated posterior moments and locations), are superimposed in an MRI of a standard brain in MNI space (d).

Given this connectivity graph, specified in terms of its nodes and connections, we tested three models. These models differed in the connections which could show putative learning-related changes, i.e., differences between listening to standard or deviant tones. Models F, B and FB allowed changes in forward, backward and both forward and backward connections, respectively (Fig. 1). All three models were compared against a baseline or null model. The null model had the same architecture described above but precluded any coupling changes between standard and deviant trials.

## Results

### Event-related potentials

**Fig. 2:**
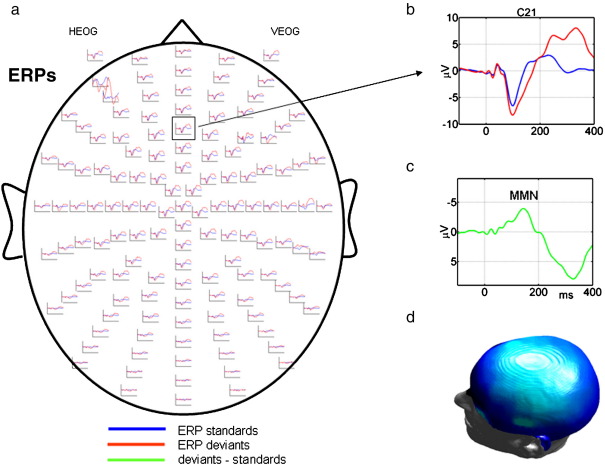
Grand mean ERPs, i.e., averaged over all subjects. (a) ERP responses to the standard and deviant tones overlaid on a whole scalp map of 128 EEG electrodes. (b) ERP responses to the standard and deviant tones at channel C21 (fronto-central). (c) MMN, the difference wave obtained by subtracting grand-average ERP to standards from ERP to deviants, at channel C21. (d) grand mean MMN response averaged across subjects and over the time window of [100, 200] ms interpolated for a 3D scalp topography.

The difference between the ERPs evoked by the standard and deviant tones revealed a standard MMN (Fig. 2). This negativity was present from 90–190 ms (Figs. 2a–c) and had a broad spatial pattern, encompassing electrodes previously associated with auditory and frontal areas (Fig. 2d).

### Model selection

**Fig. 3:**
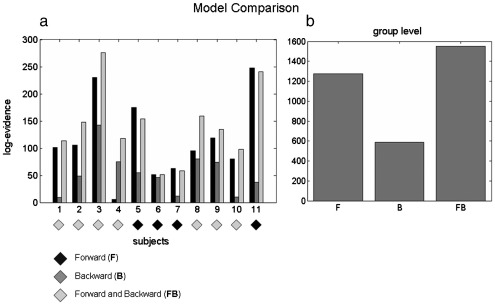
Bayesian model selection among DCMs for the three models, F, B and FB, expressed relative to a DCM in which no connections were allowed to change (null model). The graphs show the free energy approximation to the log-evidence. (a) Log-evidence for models F, B and FB for each subject (relative to the null model). The diamond attributed to each subject identifies the best model on the basis of the subject's highest log-evidence. (b) Log-evidence at the group level, i.e., pooled over subjects, for the three models.

Four different DCMs, forward only (F-model), backward only (B-model), forward and backward (FB-model) and the null were inverted for each subject. Fig. 3 illustrates the model comparison based on the increase in log-evidence over the null model, for all subjects. Fig. 3a shows the log-evidence for the three models, relative to the null model, for each subject, revealing that the three models were significantly better than the null in all subjects. The diamond attributed to each subject identifies the best model on the basis of the highest log-evidence. The FB-model was significantly better in 7 out of 11 subjects. The F-model was better in four subjects but only significantly so in three (for one of these subjects [subject 6], model comparison revealed only weak evidence in favour of the F-model over the FB-model, though still very strong evidence over the B-model. In all but one subject, the F and FB-models were better than the B-model. Fig. 3b shows the log-evidences for the three models at the group level (i.e., using. Eq. (4)). Both F and FB are clearly more likely than B and, over subjects, there is very strong evidence in favour of model FB over model F.

**Fig. 4:**
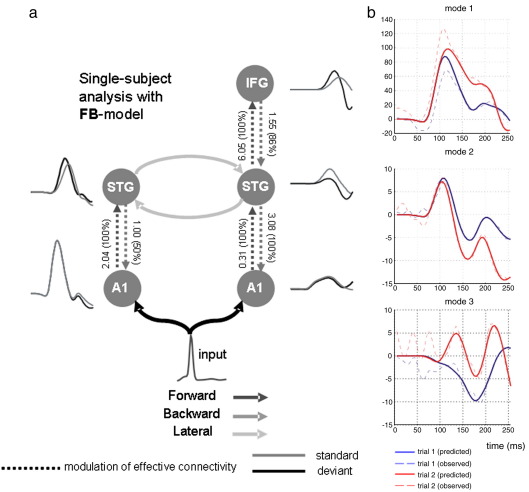
DCM results for a single subject [subject 9] (FB model). (a) Reconstructed responses for each source and changes in coupling adjacent to the connections during oddball processing relative to standards. The mismatch response is expressed in nearly every source. (b) Predicted (solid) and observed (broken) responses in measurement space, which result from a projection of the scalp data onto their first three spatial modes.

Fig. 4a shows, for the best model FB, the predicted responses at each node of the network for each trial type (i.e., standard or deviant) for a single subject (subject 9). The coupling gains and the conditional probability of the gains being greater or smaller than one are shown for each connection in the network. The values on each connection represent a scaling effect, for example, a coupling change of 2.04 from lA1 to lSTG in model means that the effective connectivity increased 104% for rare events relative to frequent events. The response, in measurement space, of the three principal spatial modes is shown on the right (Fig. 4b). This figure shows a remarkable agreement between predicted (solid) and observed (dotted) responses.

**Fig. 5:**
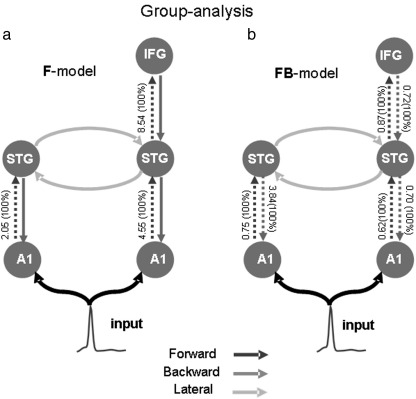
Coupling gains and their conditional probability estimated over subjects for each connection in the network for models F (a) and FB (b). There are widespread learning-related changes in all connections, expressed as modulations of coupling for deviants relative to standards.

Fig. 5 summarises the conditional densities of the coupling parameters for the F-model (Fig. 5a) and FB-model (Fig. 5b). The coupling gains and the conditional probability of the gains being greater or smaller than one, pooled over subjects, are shown for each connection in the network (i.e., using Eq. (6)). For the F-model the effective connectivity has increased in all connections with a conditional probability of 100%. For the FB-model the effective connectivity has changed in all forward and backward connections with a probability of 100%. Equivalently, and in accord with theoretical predictions, all extrinsic connections (i.e., influences) were modulated for rare events as compared to frequent events.

## Discussion

This study evaluated the predictive validity of DCM by looking at its reproducibility over subjects in the context of the MMN. To this end we used DCM to explain differences in ERPs in terms of changes in effective connectivity. For each subject we used three models of connectivity, within the same underlying cortical architecture but differing in modulations of specific types of connections. There was a very reproducible pattern of results across subjects. Model comparison revealed that conjoint changes in forward and backward connections (FB-model), relative to changes in forward (F-model) or backward connections alone (B-model) are consistently better across subjects. In every subject, these models were better than a null model that precluded any coupling changes. The evidences for models F and FB were, overall, much bigger than the model evidence for B. A similar consistency was observed quantitatively, in terms of the conditional densities of each connection; as predicted, the coupling estimates change between the two event types, i.e., standards and deviants.

In all but one subject the F-model was better than the B-model. This is an important result because both of these models had the same number of parameters. This means that any difference in the model evidences can only be explained by their ability to predict the observed response. The probability that 10 out of 11 comparisons would select the same model, by chance is exceedingly small. This suggests the DCM is sensitive to a systematic difference in ERPs to oddballs and standards and that difference, formally, lies in the network architectures used to model the observed responses. Furthermore, the FB-model was significantly better than both, in 7 out of 11 subjects. This is another important result because it shows that a more complex model (that can fit the data more accurately) is not necessarily the most likely model.

### Choice of paradigm

We have deliberately chosen a paradigm that evokes two responses exhibiting a large difference over peri-stimulus time. Note that this is not a classical oddball paradigm, as employed in the MMN literature. The large pitch difference between responses to standards and deviants elicits large differences for both N1 and MMN components, which cannot be disentangled. In this sense, this paradigm is not necessarily appropriate for modelling the MMN alone, but suitable for assessing the reproducibility of DCM: DCM is a model of dynamic responses or transients that are continuous in time. This means that the DCM is not an explanation for a particular response component (e.g., the MMN) but the compound response over all peristimulus times; it is likely that our paradigm induced N1 effects (due to the large different in standard and oddball tones) and, at least phenomenologically, a P300-like component (although the analysis presented here only went up to 250 ms). However, all these components could be explained by differences in a simple network model of interacting neuronal populations. It will be interesting to assess the ontological status of response components (e.g., N1, MMN, and P300) in the light of mechanistic models like DCM (see below).

### Choice of model

DCM is not an exploratory technique; it does not explore all possible models: DCM tests specific models of connectivity and, through model selection, can provide evidence in favour of one model relative to others. The results of a DCM analysis depend explicitly upon the models evaluated, which are generally motivated by mechanistic hypotheses. This means there may exist other equally or more plausible models with different architectures (in respect of the areas and connections involved). The network we chose for our DCMs was motivated by the results of previous MMN (Opitz et al., 2002; Doeller et al., 2003). It has been shown that the generators of the MMN lie bilaterally on the temporal cortex. In addition, some studies have shown bilateral generators on the frontal cortex, which are activated later than the auditory cortex generators (Rinne et al., 2000). Recent studies showed a double peak over frontal scalp locations suggesting the existence of two sub-components for the MMN (Opitz et al., 2002 and Doeller et al., 2003). The early component is reported to peak around 90–120 ms and can be modelled with sources located bilaterally in the superior temporal gyrus (STG). Components within peaking around 140–170 ms have been shown to be modelled effectively with dipoles in both left and right inferior frontal gyrus (IFG) that are usually stronger in the right hemisphere. Moreover, the right IFG is reported more consistently as in the literature than the homologous source on the left. This is why we used a unilateral right IFG. The inclusion of both left and right primary auditory cortices was necessary because DCM attempts to explain each ERP individually and any differences (in this case, responses to standards and deviants and the implicit mismatch). Therefore, left and right A1 were chosen as the cortical targets of thalamic input, for processing the auditory information.

Indeed there may be other plausible models. *So, why not include more sources in the model, for example left IFG?* This is an important question that has a principled answer. Each model is defined by its number of sources and their connectivity. Given a particular model, DCM will optimise the parameters of that model. To explore the space of models, one uses the marginal likelihood or log-evidence to compare one model with another. This enables one to adjudicate between different models with different number of sources. This is potentially an important application of DCM in the context of model selection. In this paper, we focussed on reproducibility of different connectivity architectures. In our next paper, we will present an exploration of model space.

### Mechanisms of ERP generation

The mechanism of MMN generation is unknown. Two sorts of hypotheses have been suggested. Traditionally, this response is thought to be associated with an automatic cortical change-detection process, which detects a difference between the current and the preceding input. It has been proposed that the MMN would reflect modifications to existing parts of a model of the acoustic environment. This model adjustment hypothesis discusses the existence of a dynamic system of change detection which updates its model of sensory input as the changes occur (Winkler et al., 1996; Sussman and Winkler, 2001). Doeller et al. (2003) suggested that the prefrontal cortex is involved in a top-down modulation of the deviance detection system in the temporal cortices. The traditional interpretation has been criticised by Jääskeläinen et al. (2004) who proposed that the MMN results from an *adaptation* mechanism (see also May et al., 1999) and is erroneously interpreted as a separate component generated by change-specific neurons. The N1 response to standard (or ‘non-novel’) sounds is thought to be delayed and suppressed (or *attenuated*) as a function of its similarity to the preceding auditory events, reflecting short-lived adaptation of auditory cortex neurons. Under this hypothesis, the response termed as MMN, would therefore be a product of an N1 differential wave emerging in the subtraction of the standards from the deviant’s ERP. Recently, the MMN has been framed within a predictive coding scheme, interpreted as a failure to suppress prediction error, which can be explained quantitatively in terms of coupling changes among cortical regions (Friston, 2005). This predicts the adjustment of a generative model of current stimulus trains (*cf*., the model adjustment hypothesis) using plastic changes in synaptic connections (*cf*. the adaptation hypothesis).

### Frequently asked questions

In presenting this work to our colleagues and for peer review a number of key questions arose. In what follows, we summarise these questions and attempt to answer them briefly.

*What is the relationship between “inverting” a DCM and inverting a classical electromagnetic forward model to locate intra-cranial source locations?* Model inversion is used in exactly the same way in both contexts. In fact, inverting a DCM subsumes the inversion of a conventional forward model. This is because DCM has two components; a neural-mass model of the interactions among a small number of dipole sources and a classical electromagnetic forward model that links these sources to extra-cranial measurements. Inverting the DCM implicitly optimises the location and orientation of the sources. Indeed, if we removed the neuronal part of the model, DCM would reduce to a conventional forward model. The current implementation of DCM for ERPs uses the electromagnetic forward model solutions encoded in the fieldtrip software (http://www2.ru.nl/fcdonders/fieldtrip).

In this paper we used informative priors on source locations. *How important are prior source locations in DCM?* Not very important; in other words, changing the location priors would not change the results very much. This is because there is very little information in the EEG or MEG measurements about the spatial location of sources. In contrast, there is an enormous amount of information about their orientation. A usual heuristic is to imagine that one is trying to infer the location and orientation of a pen-torch that is illuminating the inside of a balloon. When looking at the balloon from outside, a small change in the position of the torch will have only a small effect on the pattern of illumination. Conversely, changing its orientation will have a large impact. In brief, location is not an important quantity, whereas orientation is. For this reason, we put relatively informative priors on location but leave the orientation parameters free. It is relatively straightforward to assess the impact of different location priors using model selection.

Here we used three eigenmodes to invert the DCM. *Does DCM deal only with three channel data?* No, DCM will fit any number of channels or modes. For computational reasons, the channel data are projected onto their principal eigenmodes to reduce the size of the matrices the inversion scheme has to handle. Generally, one would use the same number of modes as there are sources. This is because data generated by an *n*-source model can only span an *n*-dimensional subspace of sensor space. Provided the signal is large, relative to noise, the first *n* principal eigenvariates should capture the majority of signal.

*What does DCM actually operate on, the channel data or some estimate of source dynamics?* DCM operates on the channel data in the same way as sources are reconstructed in a conventional setting. In both cases, one needs to specify the number of sources and how their activity is expressed in sensor space. The only difference is that, with DCM, one also has to specify the connections among the sources and which sources receive sensorimotor input.

*How is the significance of the difference between models evaluated at the between-subject level?* For a single subject, a difference in log-evidence of about three is considered strong evidence in favour of the more likely model. This is because a difference of three means the evidence for the more likely model is about 20 times the evidence for the other. This can be compared to the use of *p* = 0.05 = 1/20 in classical inference. When pooling log-evidences from several subjects, one adds the evidences and, implicitly the differences. This is because adding logs is the same as multiplying probabilities and the probability of getting two independent sets of data is simply the product of the probabilities of getting each alone. It can be seen that one will quickly reach a threshold of three if, and only if, the difference in log-evidences is consistent over subjects.

*How does one reconcile different results from different DCMs?* In this paper, we have looked at the reproducibility of DCMs in terms of consistency over subjects in model space. Although not the focus of this paper, it is also interesting to address the consistency of the model parameters. Inference on the parameters of a particular DCM is conditional upon the specific model and data used. Clearly, one could get very different conclusions by changing the model, as we find here for F and FB models (see Fig. 5). One may be very confident about contradictory results from two different models. This contradiction is resolved by considering the relative likelihoods of the two models. Generally, one only considers inference on the parameters of the best model. It is possible to use Bayesian model averaging; however, this is most useful when the models have roughly the same probability. If the results change under the *same* model with *different* datasets, then this speaks to inter-subject variability that may be real. Clearly, it would be re-assuring to see that the parameters estimates from each model were also consistent over subjects. This can be addressed with a second, between-subject analysis of the parameter estimates. To illustrate the consistency of DCM inversion in terms of model parameters, we used a classical multivariate statistical analysis (MANOVA) of subject-specific parameter estimates, from the best model (FB). In this analysis, we summarised the interesting DCM parameters using: the average coupling changes for the forward connections and the average changes for backward connections. Using just two dependent summary variables ensured we had sufficient (i.e., nine) degrees of freedom to infer the group averages where significantly different from zero. The resulting *F*-value (based on Wilk's lambda; *df* 2,9) was 4.725 corresponding to a *p*-value of 0.04. This implies a systematic and consistent pattern of changes over forward and backward connections, over subjects. The average change in forward connections was 80% and the average change in backward connections was 57%. This is in conformity with the average change in forward and backward connections for the DCM analysis of grand mean data, which was 42% and 52% respectively. A more informed way of addressing this issue would involve a hierarchical Bayesian model that includes random effects from each subject. Thus will be focus of future work.

## Conclusion

In this paper, we have looked at possible mechanisms underlying the generation of the MMN at the level of coupling among sources, and the extent to which the ensuing inferences generalise over subjects. Our specific model comparison addressed hierarchical implementations of predictive coding, in terms of extrinsic forward and backward connections. Testing the adaptation hypothesis would require modulations of intrinsic connections (i.e., among neural subpopulations within cortical units). In a subsequent paper we will look at the relative roles of extrinsic and intrinsic connectivity changes (Kiebel et al., 2007).

In summary, this study suggests that the emergence of the MMN can be explained by repetition-dependent change in forward and backward connections. This was found consistently across the group of subjects studied. Changes in forward connections might be associated with the construction of higher-level representations of sensory data. This is consistent with our conjecture that the MMN reflects perceptual learning of standards, using predictive coding. Prediction error, defined as the difference between the input observed and that predicted by a generative model and inferred causes, is minimised in predictive coding schemes. Under this framework, forward connections provide feedback by conveying prediction error to higher levels and backward connections provide contextual guidance to lower levels. Hence, the MMN could represent a failure to predict bottom-up input and consequently a failure to suppress prediction error. This might be expressed as apparent increases in bottom-up influences when stimuli are unpredicted or deviant. Here we have shown that DCM for ERPs/ERFs behaves consistently across subjects and presented the first attempt to invert a mechanistic model for the MMN using empirical data.

## Figures and Tables

**Table 1:** Prior coordinates for the locations of the equivalent current dipoles in MNI space (mm)

Left primary auditory cortex (lA1)	− 42, − 22, 7
Right primary auditory cortex (rA1)	46, − 14, 8
Left superior temporal gyrus (lSTG)	− 61, − 32, 8
Right superior temporal gyrus (rSTG)	59, − 25, 8
Right inferior frontal gyrus (rIFG)	46, 20, 8
